# Annotations

**Published:** 1889-07-27

**Authors:** 


### Annotations.

**Weariness.**

A tramp knows what it is to be leg-weary, a farm labourer to be body-weary, a literary man to be brain-weary, and a sorrowing man to be soul-weary. The sick are often weary, even of life itself. Weariness is generally a physiological "ebb-tide," which time and patience will convert into a "flow." It is never well to whip or spur a worn-out horse, except in the direst straits. If he mends his pace in obedience to the stimulus, every step is a drop drawn from his life-blood. Idleness is not one of the faults of the present age; weariness is one of its commonest experiences. The cheques that many a man draws on his physiological resources are innumerable; and as these resources are strictly limited, like any other ordinary banking account, it is very easy to bring about a balance on the wrong side. Adequate rest is one kind of repayment to the bank, sound sleep is another, regular eating and good digestion another. One day's holiday in the week and one or two months in the year for those who work exceptionally hard usually bring the credit balance to a highly favourable condition; and thus with care and management physiological solvency is secured and maintained. But a physiological fortune is as good a thing, or even a better thing, than a money fortune. Stored resources, well invested, keep the mind easy and the body youthful. If, however, a man have not these, but only enough of strength to go on steadily from day to day, he should watch carefully against excessive weariness. A feeling of prostration is the dark thunder-cloud that portends a change in the atmosphere. Health, like weather, may "break"; and when once it is broken nobody knows when the barometer will mark "set fair" again. Weariness coming on in the ordinary course of work, without any special and temporary cause, is Nature's demand for an immediate holiday. The horse is tired. He does not want the whip, but a month's run in a quiet and abundant pasture. As nothing in the world can properly satisfy hunger except food, so no drug or stimulant of any kind except rest can restore the weary to energy and health. The doctor's tonic is a very good thing in its way, but it will no more act as a substitute for rest than a glow-worm's light will serve the same purpose as the moon.

**"A Reasonably Dressed Woman!"**

A happy phrase, but an impossible ideal! What is dress for? "To clothe the form," answers Mr. J. C. Horsley, R.A. That is the artist's view of the matter, and it agrees in all essential respects with the doctor's, only it is more poetical, more artistic. Dress, the doctor considers, is to protect the body against changes of temperature. The artist admits that, but adds that it should ordinarily be so constructed as to show to advantage the grace and beauty of the human form. On behalf of our fellow physicians we willingly enlarge our conception of dress so as to include the artist's entirely rational view. "Nothing is more charming to see than a reasonably dressed woman," says Mr. Horsley. This is a decidedly universal affirmative, and it is possible to imagine that many women might take exception to it on the ground that to them a still more charming sight is a reasonably dressed man. But there need be no quarrel on that point; to a man of rational mind, and soul imbued with even the smallest modicum of poetry, a reasonably dressed woman, other things being equal, is a sight as charming as it is rare. Mr. Horsley says that as a subject painter he has had all his life a great deal to do with dress-cutting—ladies' dress-cutting of course. Unhappy man! If he has not committed suicide in despair of his fruitless labours he must have a highly pachydermatous nervous system. It is not, however, that he has not "felt," but he seems to have the power of relieving his mind by good, vigorous railing, not to say "swearing." He speaks not of the vagaries, but of the "atrocities" of fashion. Crinolines and dress-improvers are, he says, among the most "atrocious things" ever invented. When it is remembered that racks, thumbscrews, red-hot torturing-irons, and comic newspapers have been invented, this statement will be considered fairly strong. A "dress-improver" he calls a "dreadful thing," fit only for a tinker's wife with a large family to carry her superfluous infant upon; or, in case of necessity, it may be allowed to have a certain use as a bracket-holder for a paraffin lamp. That "horror of horrors," a small waist, he would not permit himself so much as to touch upon, further than to say that it always gave him a "pain at the heart." Alas, Mr. Horsley! If you have made up your mind to start a crusade against stupid and ridiculous ladies' fashions, you deserve the pity of all sane men. We speak from experience. We have tried reasoning, pleading, laughing, scolding—but all to no purpose. A fashionable woman is really sublime. If a church-steeple headdress be in fashion to-day, a church-steeple she will have on the top of her head, though she be seven feet high in her stocking soles; if a wooden breadplate be *de rigeur* to-morrow, a wooden breadplate must be flattened down on her wondrous skull, even though her height be such that her husband's elbow is beyond her reach. If the doctor has failed, when his discourse has been strengthened by the most harrowing tales of lost health, fainting, sudden death, and we know not what, how may the mere artist hope to succeed? It is possible, however, that an appeal to the artistic sense may be more powerful than an appeal to reason or love of life. If women can once be made to see that reasonable dress is more truly beautiful than any other, the day will be won.

**Would-be Suicides.**

How are attempts at suicide like that of the young actress whose name was in everybody's mouth a few days ago to be prevented? It is probable that the humiliation of appearing in a police-court, and the general consciousness that she has made a fool of herself, may exercise a salutary influence on Miss Mabel Love; if, on the other hand, the publicity given to her escapade may not lead her to gratify in some other foolish or criminal fashion her taste for notoriety. But suicide she will not try again, unless, indeed, she is really suffering from insanity; she knows what it is like. Suicide is a romantic dream of many morbid young girls, and some of the weaker of them indulge in attempts at it without any real determination to take their own lives. They rather want to draw attention to themselves and their more or less imaginary woes; and in many cases take a certain amount of precaution that the attempt shall be perceived, and its result prevented. With regard to drowning in the Thames, "Blackfriars Bridge if they do mean it, London Bridge if they don't," is the dictum of a friend of ours, not cold-hearted, but eminently sensible; and it is needless to say that a very large number of the abortive attempts at suicide that are recorded in the daily papers are made from London Bridge. The would-be self-destroyers are not often in despair; they have not been hopelessly beaten in the struggle for existence; they do not want bread; neither do they want companionship, love, sympathy—the food which the soul requires as much as the body needs bread. If they are isolated, either in fact or fancy, they have chosen their isolation; they have withdrawn from the natural ties of kinship of their own free will, and too often have withdrawn from its duties as well. It cannot be too strongly said, now that she is made the heroine of so many novels, and in consequence is becoming so common in real life, that the *femme incomprise* is a nuisance, and very often a vain, selfish, and ill-tempered nuisance. When she takes to attempting suicide she becomes a public nuisance as well as a family disgrace, and as such ought to be suppressed. In New York a great reduction in attempts at suicide, especially among women, has been effected by making it a criminal offence, with heavy penalties attached to it. It may be romantic to die by your own hand, or even to be rescued and fussed over before life is extinct, but there is no romance in being sent to prison for two years, with the option of a heavy fine, for an unsuccessful attempt at suicide.

